# Treatment adherence across different psychiatric disorders: findings from a large patient cohort

**DOI:** 10.1192/j.eurpsy.2024.575

**Published:** 2024-08-27

**Authors:** N. Girone, B. Benatti, M. Cocchi, F. Achilli, C. Viganò, M. Vismara, B. Dell’Osso

**Affiliations:** ^1^Department of Mental Health, Sacco University Hospital; ^2^Center for Neurotechnology and Brain Therapeutic, “Aldo Ravelli” Center, University of Milan, Milan, Italy; ^3^Department of Psychiatry and Behavioral Sciences, Bipolar Disorders Clinic, Stanford University, California, United States

## Abstract

**Introduction:**

Medication adherence was defined by the WHO as “the extent to which a person’s behavior coincides with the medical advice given” (WHO, 2003). Existing literature indicates that approximately 49% of patients with major psychiatric disorders do not fully adhere to their prescribed psychopharmacological therapy (Colom et al, 2002). Non-adherence can lead to partial therapeutic responses or treatment resistance, increased risk of relapse, re-hospitalization, elevated suicide risk, and overall poorer functioning, thereby compromising the patient-doctor therapeutic relationship (Garcìa et al, 2016).

**Objectives:**

The aim of the present study was to assess potential differences in terms of clinical features related to adherence to treatment in a large cohort of psychiatric patients of an Italian psychiatric department.

**Methods:**

The study included 307 psychiatric patients, of any gender or age, diagnosed with unipolar depression (UD), bipolar depression (BD), anxiety disorders (AD), schizophrenic spectrum disorders (SS) or a primary diagnosis of personality disorders (PD), based on DSM-5 criteria. Patients were consecutively recruited from the Department of Psychiatry at Luigi Sacco University Hospital, in Milan. The patient’s adherence to treatment was evaluated using the Clinician Rating Scale (CRS), with a cut-off of ≥ 5 defining adherence subgroups (A+: score ≥ 5; A-: score < 5). Comparative and predictive analysis were performed for the whole sample and the two adherence subgroups.

**Results:**

Overall, nearly one-third of the whole sample reported suboptimal medication adherence. Specifically, rates were approximately 35.3% and 32.7% for BD and SS, respectively, followed by 30.8% for PD, 28% for AD and, 20.3% for UD (see Figure 1). Patients with A- showed significantly higher current substance abuse (17.8% vs 4.5%, p<.001), along with a higher rate of lifetime substance abuse, although with a trend towards significance (31.5% vs 20.5%; p=.057). Moreover, the A- group had a significantly higher number of lifetime hospitalizations (1.35 ± 1.8 vs 0.73 ± 1.11; p<.001) and higher rate of previous psychotropic treatment dropouts compared to the A+ group (90% vs. 36.2%; p<.001, see Figure 2).

**Image:**

**Figure:**
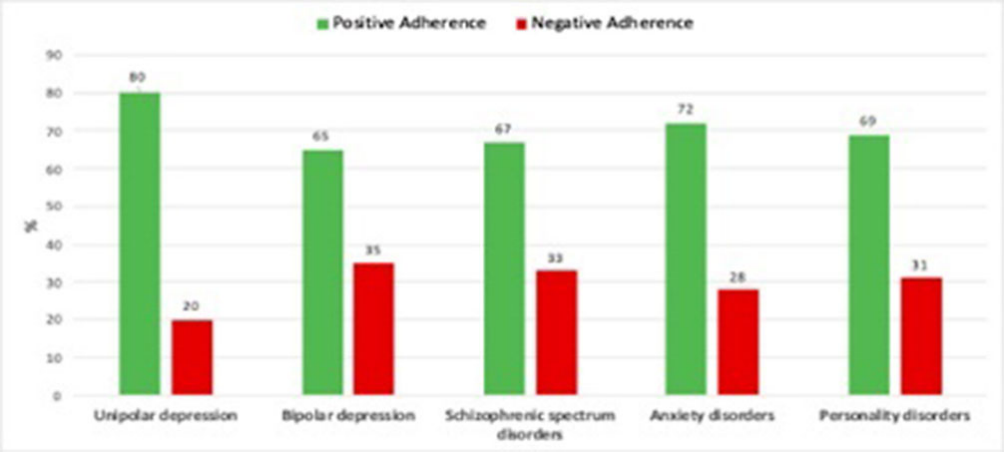

**Image 2:**

**Figure:**
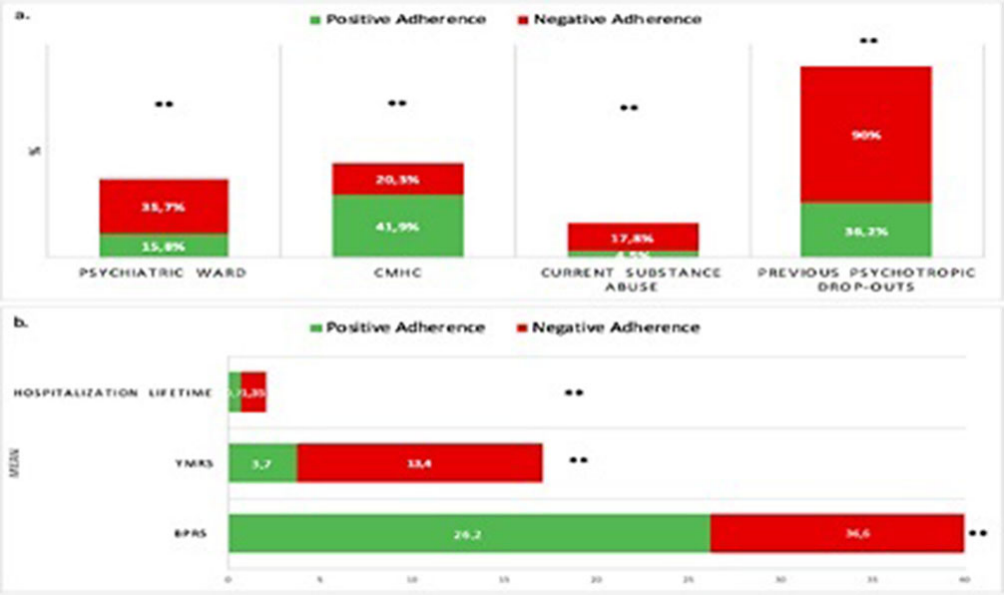

**Conclusions:**

Approximately one-third of the whole sample reported a suboptimal medication adherence, with varying rates across different diagnoses. Current and lifetime substance abuse appears to be an unfavorable transdiagnostic factor. Additionally, severe outcomes such as increased hospitalizations and a more acute disease presentation are linked to poorer adherence. Recognizing the characteristics of adherence patterns within specific diagnostic categories is crucial for designing precise interventions to enhance patient outcomes and optimize the overall effectiveness of treatment.

**Disclosure of Interest:**

N. Girone: None Declared, B. Benatti Speakers bureau of: Angelini, Lundbeck, Janssen, Rovi., M. Cocchi: None Declared, F. Achilli: None Declared, C. Viganò: None Declared, M. Vismara: None Declared, B. Dell’Osso Grant / Research support from: Angelini, Lundbeck, Janssen, Pfizer, Otzuka, Neuraxpharm, and Livanova, Speakers bureau of: Angelini, Lundbeck, Janssen, Pfizer, Otzuka, Neuraxpharm, and Livanova

